# Chinese herbal extract granules combined with 5-aminosalicylic acid for patients with moderately active ulcerative colitis: study protocol for a multicenter randomized double-blind placebo-controlled trial

**DOI:** 10.1186/s13063-020-05012-8

**Published:** 2021-01-13

**Authors:** Zhaofeng Shen, Kai Zheng, Jiandong Zou, Peiqing Gu, Jing Xing, Lu Zhang, Lei Zhu, Hong Shen

**Affiliations:** 1 Institute of Digestive Diseases, Jiangsu Province Hospital of Chinese Medicine, Affiliated Hospital of Nanjing University of Chinese Medicine, Nanjing, China; 2 School of Public Health, Nanjing Medical University, Nanjing, China; 3 Department of Gastroenterology, Jiangsu Province Hospital of Chinese Medicine, Nanjing, China

**Keywords:** Ulcerative colitis, Chinese herbal medicine, 5-Aminosalicylic acid, Multicenter randomized controlled trial, Study protocol

## Abstract

**Background:**

Ulcerative colitis (UC) is an intestinal inflammatory disease characterized by inflammation of the colonic mucosa. With unknown pathogenesis, it has become a chronic lifetime disorder worldwide. In patients with moderately active UC, several therapies (e.g., aminosalicylates, corticosteroids, immunosuppressants, and biologics) are recommended for induction (or maintenance) of remission. Given the side effects and disease burden, it is difficult for most patients to achieve ideal treatment goals in clinical practice. Chinese herbal medicine (CHM), as a complementary therapy, has been widely used in the management of UC in China. Qing-Chang-Hua-Shi granule (QCHS) is a classical Chinese herbal formula. Our preliminary study suggested that the QCHS decoction has a significant effect on patients with moderately active UC. However, its effectiveness and safety has not been evaluated convincingly. Therefore, we designed this protocol to investigate the efficacy of QCHS granule for moderately active UC.

**Methods:**

This is a multicenter, randomized, double-blind, placebo-controlled, superiority trial. A total of 120 patients with moderately active UC will be recruited from 10 hospitals in China. Each eligible participant will be randomly assigned to receive QCHS granule or placebo for 12 weeks. Both groups will be given basic treatment with mesalazine (4 g/day). The primary outcomes are the clinical response (remission) rate. The secondary outcomes are health-related quality of life, endoscopic response rate, mucosal healing rate, and inflammatory markers (e.g., fecal calprotectin and CRP). The whole study period will last 36 weeks, including 24 weeks follow-up time. According to the intention-to-treat principle, variables will be assessed at 2, 4, 6, 8, 10, and 12 weeks after study commencement.

**Discussion:**

This is the first randomized controlled clinical study protocol regarding Chinese herbal extract granules in the management of moderately active UC. We aim to investigate the superiority of QCHS granules over placebo in terms of induction of remission. If the trial shows significant benefits of QCHS granules, it will help clinical practitioners, UC patients, and policymakers make more informed choices in the decision-making.

**Trial registration:**

Chinese Clinical Trial Registry ChiCTR-IOR-14005554. Registered on 27 November 2014.

**Supplementary Information:**

The online version contains supplementary material available at 10.1186/s13063-020-05012-8.

## Background

Ulcerative colitis (UC) refers to a subtype of inflammatory bowel disease (IBD), which is characterized by chronic idiopathic inflammation of the large intestine (e.g., colonic mucosa) [1, 2]. As a lifelong disease [3, 4], UC has a significant impact on health-related quality of life [5, 6]. To date, the pathogenesis mechanism of UC is not fully understood, associated with multiple factors (e.g., heredity, environment, immunity, and behavior) [7, 8]. As a result, it has become a global refractory disease with worldwide shifting epidemiological characteristics. According to a recent survey, the incidence and prevalence in developed areas such as North America and Europe have been stable [9]. However, the data in Asia and other developing countries have encountered a significant increase over the past decade [10, 11]. Currently, the optimal goal of management is to induce (steroid-free) remission, maintain remission, and prevent disease-related complication and health-related quality of life [12]. Besides, it is believed that an emerging goal in UC management is mucosal healing. To achieve these goals, 5-aminosalicylic acid, corticosteroids, immunosuppressants, biological agents, and other promising treatments (e.g., fecal microbiota transplantation) have been developed one by one [13]. As a consequence, budesonide, corticosteroids, and anti-TNF therapy (e.g., adalimumab, golimumab, and infliximab) have been strongly recommended to induce remission for moderately active UC with moderate to high quality of evidence [14]. However, many patients do not react well to these conventional drugs in clinical practice. What is worse, most of these treatments have limitations in safety and efficacy, such as serious side-effects, long course of treatment, heavy burden of disease, and so on [15, 16]. Therefore, there still seems to be room for improvement in the management of moderately active UC.

Historically, in Asia (especially in China), Chinese herbal medicine (CHM) has been widely used for UC due to the unique advantages of efficacy and safety [17]. Under the circumstance, an increasing amount of evidences have shown that CHM have potentially positive effects on UC [18–20]. According to the theory of traditional Chinese medicine (TCM), CHM plays an irreplaceable role in the management of UC.

Over the past decade, our group has been searching for Chinese herbs that can be used for UC. Through years of our clinical practice, we found that QCHS formula developed from classical Chinese herbal formulas could not only relieve patients’ clinical symptoms, but also promote mucosal healing by adjusting the balance of the body. Our previous studies in vivo have proved that QCHS was associated with significant benefits regarding ameliorating the damage to colon length, suppressing inflammatory cytokines and mediators, alleviating oxidative stress through β2AR/β-arrestin2/NF-κB signaling pathway [21, 22]. Furthermore, QCHS could significantly inhibit apoptosis in HT-29 cells through MEK/ERK signaling via SGK1 [23]. However, our preliminary studies were limited to systematic top-level design (e.g., small sample size, placebo effect). Given these findings, the evidence of its effectiveness and safety was unconvincing, especially in patients with moderately active UC. Under the circumstance, we designed this protocol, a prospective, multicenter, randomized, double-blind, placebo-controlled, superiority trial, to further determine the efficacy and safety of QCHS granule combination therapy for UC patients who do not respond to 5-ASA after 4 weeks. We predict that patients with moderately active UC will benefit in terms of clinical remission, mucosal healing, and quality of life.

## Hypothesis and objective

In our protocol, we hypothesize that QCHS granule combined with basic treatment (5-ASA) is superior to placebo plus 5-ASA on clinical response (remission) rate, mucosal healing rate, clinical syndromes, quality of life, inflammatory mediators (e.g., fecal calprotectin, TNF-α, and hs-CRP), and adverse side-effects.

The primary objective of this trial is to investigate the efficacy of QCHS granule combined with 5-ASA in the management of patients with moderately active UC. Furthermore, the secondary objective is to explore the safety of QCHS granule for moderately active UC.

## Methods

### Study design

**Fig. 1:**
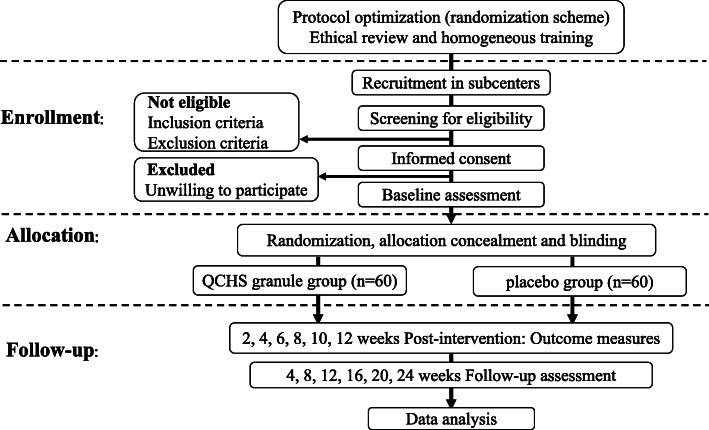
Overview (flowchart) of the protocol

**Fig. 2:**
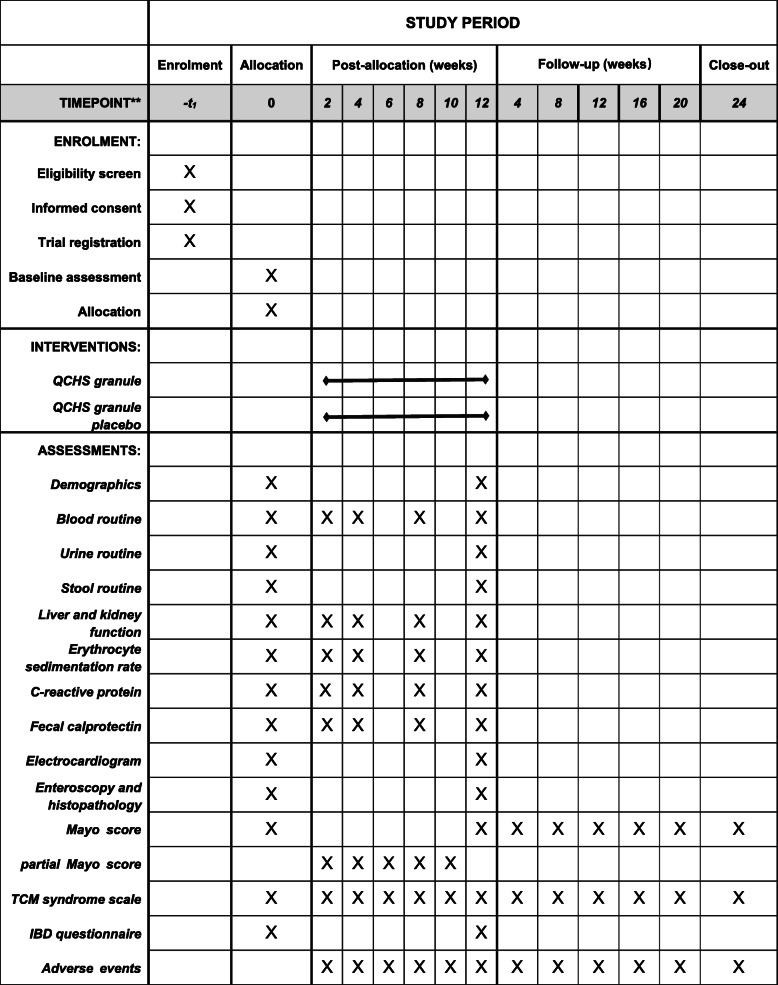
Schedule of enrolment, interventions, assessments, and follow-up

This is a prospective, multicenter, randomized, double-blind, placebo-controlled, superiority clinical trial, which conforms to the Consolidated Standards of Reporting Trials (CONSORT) 2010 statement guidelines [24], the Standard Protocol Items: Recommendations for Interventional Trials (SPIRIT) 2013 Statement [25], and the Standard Protocol Items for Clinical Trials with Traditional Chinese Medicine: Recommendations, Explanation and Elaboration (SPIRIT-TCM) Extension 2018 Statement [26]. The study began in January 2016 and will last until October 2022. A total of 120 eligible patients will be enrolled and randomized into the QCHS granule group or the placebo group. All patients will voluntarily sign the informed consent prior to enrollment. Prior to allocation, each of them will be screened by the eligibility criteria. According to patients’ included sequence number equally, block randomization will be performed to ensure equal group sizes with an allocation of 1:1 (permuted block sizes of 6). Participants in the QCHS granule group will receive QCHS granule (125 g daily, orally) for continuous 12 weeks, while patients in the placebo group will receive QCHS granule placebo (125 g daily, orally) for the same duration. Both groups will be given basic treatment with mesalazine (5-aminosalicylic acid, 4 g/day). Researchers and patients will be blinded from the beginning of the trial. All interested outcomes including patient-reported outcomes will be collected before (0 weeks) and after intervention (2 weeks, 4 weeks, 6 weeks, 8 weeks, 10 weeks, and 12 weeks). The whole study period will last 36 weeks, including 24 weeks of follow-up. The complete SPIRIT (2013) checklist and SPIRIT-TCM (2018) checklist for the study are provided in Additional files 1 and 2. The overview and the SPIRIT schedule of the study are illustrated in Figs. 1 and 2, respectively.

### Study setting

This study was developed by a research team in Nanjing (Jiangsu Province Hospital of Chinese Medicine) together with nine teams in China. The collaborators were selected based on the characteristics of sites as well as the strength and experience of their teams. As a result, patients will be recruited from 10 subcenters (tertiary hospitals) across China, including Affiliated Hospital of Nanjing University of Chinese Medicine, Beijing Hospital of Chinese Medicine, LongHua Hospital Shanghai University of Traditional Chinese Medicine, Guangdong Province Hospital of Chinese Medicine, The First Affiliated Hospital of Henan University of Chinese Medicine, ShengJing Hospital of China Medical University, Affiliated Hospital of Shanxi University of Chinese Medicine, The Second Affiliated Hospital of Fujian Traditional Chinese Medical University, The First Affiliated Hospital of Heilongjiang University of Chinese Medicine, and Nantong Hospital of Chinese Medicine. Each research institution will follow the same research protocol.

### Ethics and registration

The protocol has been approved by the Ethics Committee of Affiliated Hospital of Nanjing University of Chinese Medicine (approval number:2014NL-074-02, Additional file 3). Each subcenter applied for local institutional review boards (IRBs) approval. Furthermore, this trial has been registered on the Chinese Clinical Trial Registry (URL: http://www.chictr.org.cn/, No. ChiCTR-IOR-14005554).

### Sample size and power calculation

The sample size was calculated according to the primary outcome (clinical response rate) at 12 weeks after initiation. The sample size calculation was based on the comparison of the proportions in the QCHS granule group versus the placebo group. On the basis of previous study and clinical experience, we predicted that the clinical remission rates at 12 weeks would be 79% in the QCHS granule group and 41% in the placebo group [27, 28]. A difference of 10% was selected as the smallest difference that would be of clinical significance. For a two-sided significance level of 0.05 (*α* = 0.05), it is estimated that a sample size of 54 subjects per group will be required to detect the superiority of QCHS granule over placebo with a test power of 80%. Under the hypothesis that some patients are unable to follow-up or unsuitable for analysis, the sample size will be inflated. Considering 10% drop out, the total planned sample size will be 60 per group.

### Randomization, allocation concealment, and blinding

A total of 120 patients with moderately active UC will be randomized into the QCHS granule group or the placebo group with an allocation ratio of 1:1. Participants will be recruited from 10 hospitals in China by centralized computer-generated software. To ensure balance by study site, a stratified and block randomization algorithm was used and randomization was stratified by study site (12 cases in each center) and block sizes of six were used within each strata. The randomization allocation sequence generated by SAS 9.4 (SAS Institute Inc., Cary, NC, USA) was converted into unique serial numbers for each enrolled subject in subcenters. QCHS granule and placebo granule will be provided to each subcenter in advance, encoded and labeled with the above serial number. To ensure blinding, the QCHS granule and placebo granule will be identical in all aspects (e.g., appearance, size, color, smell, taste, containers, and doses). Participant and Principal Investigators will be blinded to group allocation throughout the research. The randomization procedure was conducted by an independent research assistant who is not involved in clinical observation or assessment. Under the supervision of Data Safety and Monitoring Board (DSMB), the randomization code will only be broken due to adverse events and statistical analysis.

### Participants

The participants will be 120 patients with moderately active UC diagnosed as per histologic and endoscopic criteria. The diagnostic criteria are based on the Second European evidence-based Consensus on the diagnosis and management of ulcerative colitis in 2012 [29] and Chinese Association of Integrative Medicine Guidelines for Diagnosis of UC in 2010 [30].

### Inclusion criteria

Patients will be included if they meet the following criteria:

1. Confirmed diagnosis of moderately active UC after taking 5-ASA for more than 4 weeks (total Mayo score between 6 and 10, endoscopy subscore > 2) [31]
2. Confirmed diagnosis of TCM syndrome differentiation (large intestine damp-heat syndrome) [30]
3. Males or females with age range between 18 and 50 years
4. Ethical principle who voluntarily sign the informed consent form

### Exclusion criteria

Patients will be excluded if they have the following criteria:

1. Patients with other intestinal diseases (e.g., bacterial dysentery, intestinal tuberculosis, Crohn’s disease)
2. Patients with serious complications (e.g., intestinal perforation, intestinal obstruction, toxic megacolon, colorectal cancer)
3. Patients who are pregnant, breast-feeding, or preparing for pregnancy
4. Patients with significant cardiac disease, hepatic disease, pulmonary disease, renal disease, and other serious diseases (abnormal urine protein, platelet value of less than 100 × 109/L, alanine aminotransferase level above the upper limit of normal, leukocyte count of less than 4.0 × 109/L)
5. Patients who have severe physical disability (e.g., blindness, deafness, dumbness, intellectual disability, mental disability, physical disability)
6. Patients who have a history of alcohol or drug abuse
7. Patients who have a history of food allergy or drug allergy
8. Patients who are participating in other clinical trials

### Withdrawal criteria

Patients who *do not* meet the inclusion criteria, have no data after randomization, have never used research medication, and take forbidden drugs will be withdrawn from this study.

### Dropout criteria

Patients who are unable to be followed-up after informed consent and randomization will be considered dropout of our study.

Those patients who discontinue will be followed up for detailed reports throughout the study. We will try our best to contact subjects, record reasons, and complete assessment. Those patients who suffer an adverse event will be treated according to clinical practice guidelines. All the data of the research process will be recorded timely and accurately.

### Termination criteria

Patients will be terminated if they meet the following criteria:

1. Abnormality of safety index during treatment (ALT more than twice the upper limit of normal, Cr more than the upper limit of normal, platelet count less than 50 × 109/L, white blood cells less than 3.0 × 109/L)
2. Worse condition during the course (bloody stools more than 6 times daily accompanied by body temperature > 37.8 °C or Hb < 10.5 g/dL)

### Recruitment, screening, and enrollment procedures

Our clinical research will be conducted in accordance with the investigator’s manual, respectively. Each subcenter need to regularly discuss the protocol and its implementation so as to have a better understanding of the trial. Specialized subject recruitment advertisement was placed in the newspaper or on the social network platform. Furthermore, fliers and brochures regarding subject recruitment have been placed inside the hospital.

According to the above-mentioned inclusion and exclusion criteria, standardized screening form was implemented to identify the potential eligible participants. After the eligible subject has been confirmed, investigators (LZ, KZ, PQG, JX, HS, and LZ) will communicate with the patient face to face explaining the purpose of the trial as well as the trial procedures. Voluntarily, participants need to sign the informed consent form if they are willing to receive them. We will try our best to follow up for detailed reports throughout the study.

### Intervention

#### Basic treatment

During the intervention period, all eligible patients with moderately active UC will receive standard treatment of Mesalazine Sustained Release Granules (5-aminosalicylic acid, 5-ASA, 4 g daily) produced by French IPSEN Pharmaceutical Factory.

#### QCHS granule group

Those patients randomized to the intervention group will receive Qing-Chang-Hua-Shi granule (QCHS, 125 g daily, orally) additionally. This Chinese herbal prescription consists of several ingredients: Rhizoma Coptidis (Huanglian) 6 g, Radix Scutellariae (Huangqin) 10 g, Herba Patriniae (Baijiangcao) 15 g, Angelicae Sinensis Radix (Danggui) 10 g, Radix Paeoniae Alba (Baishao) 20 g, Radix Angelicae Dahuricae (Baizhi) 12 g, Radix Aucklandiae (Muxiang)6 g, Radix Sanguisorbae (Diyu) 10 g, Lithospermum Erythrorhizon (Zicao) 10 g, Rubia Cordifolia (Qiancao) 20 g, and Licorice (Gancao) 6 g.

#### Control group

Patients in the control group will be treated with 5-ASA plus QCHS granule placebo (125 g daily, orally). QCHS granule placebo is similar to QCHS granule in terms of appearance, size, color, smell, taste, doses, and containers. Furthermore, the composition of the placebo is starch, maltodextrin, caramel pigment, tartrazine pigment, sugar octoacetate, amaranth, and lactose.

According to the Good Agricultural Practice (GAP), all ingredients of Chinese herbal granule and placebo were manufactured by Jiangyin Tianjiang Pharmaceutical Co., Ltd., Jiangyin, China. Raw herbs were extracted in hot water, and the aqueous extract was concentrated, dried, and packed in sealed opaque packages. According to the theory of Chinese medicine, the patient should take half of the decoction in the morning and half in the evening. Instruction and code label will be tagged outside the container. Participants were asked to dissolve the granules by hot water and drink the mixture.

### Study period

The total course of intervention is 12 weeks with 24 weeks follow-up. After enrollment, the trial will last 36 weeks continuously.

### Outcomes

The primary outcome of this study is the clinical response rate in patients with moderately active UC. The clinical response refers to a decrease (from the baseline) of the total Mayo score by at least 30% (or 3 points) together with a decrease of the rectal bleeding subscore by at least 1 point (or rectal bleeding subscore of 0 or 1 point) with no individual subscore exceeding 1 point [32].

The secondary outcomes are as follows: health-related quality of life measured by the Inflammatory Bowel Disease Questionnaire (IBDQ) [33], endoscopic response rate defined as a decrease of Mayo disease activity index endoscopy subscore by at least 1 [32], mucosal healing rate defined as endoscopy subscore of Mayo disease activity index of 0 or 1, and improvements in inflammatory markers (e.g., fecal calprotectin, tumor necrosis factor-α and hypersensitive-C reactive protein, erythrocyte sedimentation rate) [34].

### Additional information

In addition, information regarding biological index (e.g., demographic characteristics, vital signs) and diagnostic index (e.g., course, status, physiological and biochemical indicators) will be obtained at baseline and follow-up. Safety index will be observed throughout the study. During the study, adverse events (AEs), such as liver dysfunction, renal dysfunction, electrocardiographic abnormality, urine protein, urine leukocyte, bloody stools, and infection, will be recorded on a standard SAE form. Those patients who experienced SAEs will be followed up for detailed reports and reported to the Institutional Review Board (IRB) of the Affiliated Hospital of Nanjing University of Chinese Medicine as required indicating expectedness, seriousness, severity, and causality.

### Data collecting and follow-up

According to the protocol, assessments will be repeated 7 times during the 12 weeks intervention: at baseline, at 2 weeks, at 4 weeks, at 6 weeks, at 8 weeks, at 10 weeks, and at 12 weeks. For those patients who have been induced to remission at the end of the intervention, we will follow up for 24 weeks. After enrollment, participants will attend a total of thirteen trial visits. Participants who completed our trial will be provided with 400 RMB for transport compensation. At the beginning and the end of the study, Mayo score, colonoscopy, mucosal histology, heath-related quality of life, biochemical parameters, and safety index will be measured. However, partial Mayo score, symptom indicators (e.g., diarrhea, bloody stools, and abdominal pain), biochemical parameters (e.g., fecal calprotectin, hypersensitive-C reactive protein, erythrocyte sedimentation rate, stool routine, occult blood test), and safety index (e.g., blood routine, liver function and kidney function) will be evaluated at 2 weeks, 4 weeks, 6 weeks, 8 weeks, and 10 weeks respectively.

### Data management and quality control

To ensure the quality of this trial, an external monitoring agency (a CRO located in Nanjing) will be hired to help data collection, management, and analysis across all subcenters. All data will be recorded (double-entered) on electronic case report forms (eCRFs) by trained investigators and will be monitored monthly via electronic data capture system. For each abnormal or missing datum, a query will be automatically sent to the investigator. Once all the inconsistencies or queries are solved, the database will be locked for statistical analysis.

### Statistical analysis

According to our protocol, all data analyses will be conducted based on pre-established statistical analysis plan. All analyses of data will be performed by SAS software (v. 9.3; SAS Institute Inc., Cary, NC, USA). To ensure the consistency and reliability of the conclusions, both intention-to-treat (ITT) analysis and per-protocol (PP) analysis will be done if necessary. All missing data will be imputed by multiple imputation. Statistical description will be performed with frequency, mean, median, standard deviation, lower quartile (P25), upper quartile (P75), minimum, and maximum. Normality of all quantitative (continuous) variables (e.g., demographic characteristics, vital signs, biochemical parameters) will be tested by the Kolmogorov-Smirnov test. For normal distribution data, independent sample *t* test and paired sample *t* test will be employed to compare parameters at the beginning and the end of the study between and within groups, respectively. For abnormal distributions data (e.g., quality of life, inflammation markers), the Mann-Whitney *U* test and Wilcoxon signed-rank test will be used instead. For qualitative (categorical) variables, the chi-square test or Fisher’s exact test will be used, such as clinical response rate, endoscopic response rate, and mucosal healing rate. In the evaluation of efficacy and analysis of influencing factors, center and disease will be used as covariates. Covariance analysis (ANCOVA) will be used [35]. Multiple linear regression or logistic regression will be used to analyze the influencing factors and evaluate the effect of gender and condition on the efficacy of the two groups. For all analyses, *P* < 0.05 will be considered statistically significant.

## Discussion

UC is a chronic immune-mediated (nonspecific) inflammatory condition. Patients with active UC are more likely to have comorbid psychological conditions of anxiety and depression, resulting in impaired social interactions or career progression [36]. As a difficult and incurable disease, the optimal goal of its management is to induce (steroid-free) remission, maintain remission (especially mucosal healing), and prevent disease-related complication and health-related quality of life [12]. Therapeutic options in patients with moderately active UC include 5-aminosalicylic acid, corticosteroids, and anti-TNF therapy (e.g., adalimumab, golimumab, and infliximab). In our clinical practice, many patients do not respond to these conventional drugs. As a result, strategies for the management of moderately active UC mainly depend on the following factors: the risk and benefit of the choice, preference of the patients, and experience of the doctors. The use of TCM in patients with moderately active UC has increased in popularity over the past decade because of the unique advantages of efficacy, convenience, safety, and low cost [18]. In Asian countries, especially in China, Korea, and Japan, herbal medicines have been widely used for approximately 2000 years to treat and manage UC-like symptoms, such as diarrhea, abdominal pain, and mucus/bloody stool. Qing-Chang-Hua-Shi granule (QCHS) is a classical Chinese herbal formula, which is composed of Huanglian, Huangqin, Baijiangcao, Danggui, Baishao, Diyu, Zicao, Qiancao, Baizhi, Muxiang, and Gancao herbs. Our previous studies have proved that QCHS could alleviate UC oxidative stress and intestinal inflammation which was related to activation of β2AR/β-arrestin2/NF-κB signaling pathway [37]. Moreover, an in-depth study found paeoniflorin had the anti-inflammatory effect in UC via inhibiting MAPK/NF-kappa B pathway and apoptosis in mice [38], and astragalus and baicalein regulated inflammation of mesenchymal stem cells by MAPK/ERK pathway [39]. Based on the abovementioned results, we explored whether QCHS could help patients with moderately active UC who do not respond to 5-ASA after 4 weeks. To the best of our knowledge, this is the first prospective, multicenter, randomized, double-blind, placebo-controlled, superiority clinical study protocol regarding Chinese herbal extract granules in the management of moderately active ulcerative colitis. Furthermore, our protocol is strictly developed according to the requirements of SPIRIT 2013 statement and SPIRIT-TCM extension 2018 statement. We hypothesize that QCHS granule combined with basic treatment (5-ASA) is superior to placebo plus 5-ASA on clinical response (remission) rate, mucosal healing rate, clinical syndromes, and quality of life. As a result, our study will be conducted by a group of experienced experts from 10 subcenters (tertiary hospitals) across China. Interactive Web Response System (IWRS) will be used throughout the study. It is inevitable that we need to control the quality of our research. If the trial shows significant benefits of QCHS granules, there is evidence that we can prescribe QCHS granule combined with 5-ASA for moderately active ulcerative colitis in the clinical practice.

### Trial status

Recruitment for the trial was planned to start on January 2016 and last until October 2022. The first patient was enrolled on April 10, 2016. The trial is enrolling patients. The current protocol is version 4.0 and is dated 20 September 2018.

## Supplementary Information

**Additional file 1.** SPIRIT 2013 Checklist: Recommended items to address in a clinical trial protocol and related documents.

**Additional file 2.** SPIRIT-TCM 2018 checklist: Recommended items for clinical trials with Traditional Chinese Medicine.

**Additional file 3.** Ethics approval (No.2014NL-074-02).

**Additional file 4.** Funding (Grant No.201407001, No.81873260).

## Data Availability

Supporting data regarding the study protocol are available as supplementary data and have been submitted alongside the main manuscript. JDZ, ZFS, LZ, and HS will have access to the final trial dataset and disclose contractual agreements. All of the individual participant data collected during the trial after deidentification will be shared. Data will be available after the trial completion and article publication. Data requestors will need to sign a data access agreement and gain access at a third party website.

## Abbreviations

CHM: Chinese herbal medicine
TCM: Traditional Chinese medicine
UC: Ulcerative colitis
IBD: Inflammatory bowel disease
QCHS: Qing-Chang-Hua-Shi granule
5-ASA: 5-Aminosalicylic acid
DSMB: Data Safety and Monitoring Board
IRBs: Institutional review boards
IBDQ: Inflammatory Bowel Disease Questionnaire
SPIRIT: Standard Protocol Items: Recommendations for Interventional Trials
FC: Fecal calprotectin
TNF-α: Tumor necrosis factor-α
Hs-CRF: Hypersensitive-C reactive protein
AEs: Adverse events
ITT: Intention-to-treat
PP: Per-protocol
