# Supplementary material for: Chinese herbal extract granules combined with 5-aminosalicylic acid for patients with moderately active ulcerative colitis: study protocol for a multicenter randomized double-blind placebo-controlled trial
Source: Trials. 2021 Jan 13;22:55. doi: 10.1186/s13063-020-05012-8 (PMC7805063; doi:10.1186/s13063-020-05012-8)
Supplement: Supplementary file 3 — Additional file 3. Ethics approval (No.2014NL-074-02). [file 13063_2020_5012_MOESM3_ESM.pdf]

伦理审查批件

批件号	2014NL-074-02		
项目名称	清肠化湿方治疗中度活动期溃疡性结肠炎的多中心临床研究		
项目来源	2014 中医药行业科研专项		
研究单位	江苏省中医院，首都医科大学附属北京中医医院，上海中医药大学附属龙华医院，广东省中医院，河南中医学院第一附属医院，福建中医药大学附属第二人民医院，中国医科大学附属盛京医院，山西中医学院附属医院，黑龙江中医药大学附属第一医院，南通市中医院中医院		
主要研究者	沈洪，朱磊		
审查类别	复审申请	审查方式	快速审查
审查日期	2014 年 09 月 23 日	审查地点	
审查委员	吴静		
审查文件	招募受试者的材料<br>修改的临床研究方案 版本号：第二版 版本日期：2014-09-20<br>修改的知情同意书 版本号：第二版 版本日期：2014-09-20		

审查意见

根据卫生部《涉及人的生物医学研究伦理审查办法（试行）》(2007)、SFDA《药物临床试验质量管理规范（2003）》、《医疗器械临床试验规定（2004）》、WMA《赫尔辛基宣言》和 CIOMS《人体生物医学研究国际道德指南》的伦理原则，经本伦理委员会审查，同意按所批准的临床研究方案、知情同意书、招募材料开展本项研究。

请遵循 GCP 原则、遵循伦理委员会批准的方案开展临床研究，保护受试者的健康与权利。研究开始前，请申请人完成临床试验注册。研究过程中若变更主要研究者，对临床研究方案、知情同意书、招募材料等的任何修改，请申请人提交修正案审查申请。发生严重不良事件，请申请人及时提交严重不良事件报告；紧急报告之后，尽快提交详细的严重不良事件随访报告。请按照伦理委员会规定的年度/定期跟踪审查频率，申请人在截止日期前 1 个月提交研究进展报告；申办者应当向组长单位伦理委员会提交各中心研究进展的汇总报告；当出现任何可能显著影响试验进行、或增加受试者危险的情况时，请申请人及时向伦理委员会提交书面报告。研究纳入了不符合纳入标准或符合排除标准的受试者，符合中止试验规定而未让受试者退出研究，给予错误治疗或剂量，给予方案禁止的合并用药等没有遵从方案开展研究的情况；或可能对受试者的权益/健康、以及研究的科学性造成不良影响等违背 GCP 原则的情况，请申办者/监查员/研究者提交违背方案报告。申请人暂停或提前终止临床研究，请及时提交暂停/终止研究报告。完成临床研究，请申请人提交结题报告。本项临床试验应当在批准之日起一年内实施，逾期未实施的，本批件自行废止。

年度/定期跟踪审查频率	请于 2015 年 09 月 23 日前 1 个月提交研究进展报告
有效期	12 个月
联系人与联系电话	吴静 025-86560515
主席签字	[signature]
伦理委员会	南京中医药大学附属医院（江苏省中医院）伦理委员会（盖章）
日期	2014 年 09 月 23 日

Translation for reference
Wu Jing
Director of IRB June.18th, 2020

Ethical Review Approval Letter

**Approval Letter No.**	2014NL-074-02		
**Title of Protocol**	Qing-Chang-Hua-Shi granules for patients with moderately active ulcerative colitis: a multicenter randomized controlled trial		
**Source of Funds**	Supported by the Special Scientific Research for Traditional Chinese Medicine of State Administration of Traditional Chinese Medicine of China		
**Research Unit**	Jiangsu Province Hospital of Chinese Medicine		
**Principal Investigator**	Hong Shen, Lei Zhu		
**Review Category**	Re-review	**Review Process**	Expedited review
**Date of Review**	2014-09-23	**Site of Review**	
**Reviewer**	Jing Wu		
**Approved Documents**	Recruitment Advertisement (1st version, 2014-08-15)<br>Revised Protocol (2nd version, 2014-09-20)<br>Revised Informed Consent Form (2nd version, 2014-09-20)		

Review Opinions

According to the ethical principles of MOH: Measures for Guidelines on Ethical Review of Biomedical Research Involving Human Subjects (2007), SFDA: Chinese Good Clinical Practice (2003), Provisions for Clinical Trials of Medical Devices (2004), WMA: Declaration of Helsinki and CIOMS: International Ethical Guidelines for Biomedical Research Involving Human Subjects, after review of IRB, the research is approved to be carried out in accordance with the approved protocol and informed consent form. Please follow the principles of GCP, follow the protocol approved by IRB to conduct the clinical research, protect the rights and well-being of subjects. Please complete the clinical trial registration before starting the research. Please submit amendment's review application, if there is any change of principal investigator, or any modification to the protocol, informed consent form, recruitment documents, etc., in the course of research. Please submit SAE report timely if SAE occurs. Please submit research progress report one month before the expiration date according to the frequency of annual/regular continuing review stipulated by IRB; please submit written report to IRB timely if there is any situation that may significantly affect the research, or increase risks to subjects. The investigator should submit non-compliance/violation/deviation report, if there is any violation of the protocol and the principles of GCP, such as the subject who does not meet the inclusion criteria or meets the exclusion criteria is enrolled; subject is not withdrew from the study when criteria for termination are met; wrong therapy or dose is given; combination therapy which is prohibited by the protocol is given; or other conditions that may adversely affect subjects' rights and interests/well-being, as well as the integrity of the research. Please submit suspension/termination report timely, if applicant suspend or prematurely terminate the clinical research. Please submit final report of research, if clinical research is complete.

**Frequency of Regular Continuing Review**	Please submit progress report one month before Sept. 23rd, 2015
**Valid Period**	12 months
**Contact Person and Phone Number**	Jing Wu 0086 25 86560515
**Signature of Chair**	Shenlin Liu
**IRB**	IRB of Affiliated Hospital of Nanjing University of Chinese Medicine (Jiangsu Province Hospital of Chinese Medicine)
**Date**	2014-09-23
