# Supplementary material for: Chinese herbal extract granules combined with 5-aminosalicylic acid for patients with moderately active ulcerative colitis: study protocol for a multicenter randomized double-blind placebo-controlled trial
Source: Trials. 2021 Jan 13;22:55. doi: 10.1186/s13063-020-05012-8 (PMC7805063; doi:10.1186/s13063-020-05012-8)
Supplement: Supplementary file 4 — Additional file 4. Funding (Grant No.201407001, No.81873260). [file 13063_2020_5012_MOESM4_ESM.pdf]

点前，依托单位须将自然科学基金委审核通过后的计划书纸质版（一式两份，应保证与电子版一致）加盖单位公章，报送至自然科学基金委项目材料接收工作组，同时将修改后的申请书纸质签字盖章页报送至相关科学部。采用邮寄方式的，请在截止日前（以发信邮戳日期为准）以快递方式邮寄，并在信封左下角注明“计划书”或“申请书签字盖章页”。请勿使用包裹，以免延误报送。报送计划书材料时，还应包括本单位报送计划书的公函和计划书清单。材料不完整不予接收。

如在规定期限内未提交和报送电子与纸质计划书或申请书纸质签字盖章页的，视为自动放弃接受资助。未按要求修改或逾期提交申请书纸质签字盖章页的，将视情况给予暂缓拨付经费等处理。

邮寄地址：北京市海淀区双清路83号项目材料接收工作组
邮编：100085
联系电话：010-62328591

附件：2018年度国家自然科学基金资助项目清单

国家自然科学基金委员会
2018年8月27日

2018年度国家自然科学基金资助项目清单（南京中医药大学）

直接费用单位：万元

单号：2018-40-0641

序号	项目批准号	负责人	申请代码	项目名称	直接费用	起止日期	资助类别/亚类说明/附注说明
67	81873189	郭盛	H2903	基于“脑-肠-菌轴”的酸枣仁多组分养心安神协同作用机制研究	56	2019.01.01-2022.12.31	面上项目
68	81873207	叶放	H2702	基于瘀热病机研究紫七软肝方对肝硬化进程中TLR4 /NF-κB通路的调控机制	25	2019.01.01-2020.12.31	面上项目
69	81873219	毕蕾	H2704	益气活血方干预DTL泛素蛋白酶途径抗NSCLC的分子机制研究	57	2019.01.01-2022.12.31	面上项目
70	81873229	樊巧玲	H2705	左归丸干预软骨-软骨下骨信号交互防治绝经后骨关节炎的效应及分子机制研究	57	2019.01.01-2022.12.31	面上项目
71	81873238	徐斌	H2707	从电针减肥效应探讨“腧穴—自主神经—脂肪”关联的特异性	60	2019.01.01-2022.12.31	面上项目
72	81873250	郑凯	H2708	清肠化湿颗粒通过Tol-DC经TGF-β/Smad/Foxp3通路诱导Treg治疗溃疡性结肠炎的机制研究	59	2019.01.01-2022.12.31	面上项目
73	81873258	方祝元	H2708	潜阳育阴颗粒调控HSF-1/Hsp90β信号网络抑制炎性纤维化反应治疗高血压肾损伤的机制研究	59	2019.01.01-2022.12.31	面上项目
74	81873259	高坤	H2708	黄蜀葵花调控NLRP3炎症小体-焦亡保护肾小管细胞的机理研究	59	2019.01.01-2022.12.31	面上项目
75	81873260	朱磊	H2708	基于代谢组学的清肠化湿方上调真杆菌调整肠道菌群影响NLRP6炎症小体治疗溃疡性结肠炎机制研究	57	2019.01.01-2022.12.31	面上项目
76	81873270	周恩超	H2708	苏茵解毒方调节肠道菌群代谢产物“解肾毒”保护肾脏的机理研究	57	2019.01.01-2022.12.31	面上项目
77	81873274	沈建平	H2708	栀子配伍丹参调控Grsf1/GPx4和Nrf2依赖性铁死亡保护氧化应激微环境下心肌的机制研究	57	2019.01.01-2022.12.31	面上项目
